# Prognostic Value of Left Atrial Strain in Heart Failure: A Systematic Review and Meta-Analysis

**DOI:** 10.3389/fcvm.2022.935103

**Published:** 2022-07-01

**Authors:** Fuwei Jia, Antian Chen, Dingding Zhang, Ligang Fang, Wei Chen

**Affiliations:** ^1^Department of Cardiology, Peking Union Medical College Hospital, Peking Union Medical College, Chinese Academy of Medical Sciences, Beijing, China; ^2^Medical Research Center, Peking Union Medical College Hospital, Peking Union Medical College, Chinese Academy of Medical Sciences, Beijing, China

**Keywords:** heart failure, left atrial strain, prognosis, systematic review, meta-analysis

## Abstract

**Background:**

Heart failure (HF) is a global health problem with high morbidity and mortality. Recently, the association between peak atrial longitudinal strain (PALS) and clinical outcomes of HF has gained increasing attention. Our aim was to systematically assess the prognostic value of PALS for adverse events in HF.

**Methods:**

PubMed, Embase, and Scopus databases were systematically searched from inception to 30 April 2022. Studies in which PALS was assessed to predict adverse outcomes in adult patients with HF were included. Study selection, quality assessment, and data extraction were performed independently by two authors. The primary endpoints were all-cause death and cardiac hospitalization.

**Results:**

Among 7,787 patients in 17 included studies, 3,029 (38.9%) experienced the primary endpoint. Patients with events had lower PALS than those without events [weighted mean difference (WMD) 6.17, 95% confidence interval (CI) 3.09–9.26, *p* < 0.001]. Each unit increment of PALS was independently associated with decreased risk for the primary endpoint [hazard ratio (HR) 0.96, 95% CI 0.94–0.98, *p* < 0.001]. The addition of PALS significantly improved the predictive power of conventional risk models [net reclassification index (NRI) 0.22, 95% CI 0.06–0.39, *p* = 0.008].

**Conclusion:**

Peak atrial longitudinal strain was an independent predictor for all-cause mortality and cardiac hospitalization in patients with HF, highlighting the clinical importance of left atrial (LA) deformation in the prognosis of HF.

**Systematic Review Registration:**

[www.crd.york.ac.uk/prospero/], identifier [CRD42020185034].

## Introduction

Heart failure (HF) is becoming a global health problem due to its high prevalence and incidence (1). Despite advances in diagnosis and treatment strategies, patients with HF still have a substantial risk for adverse clinical events, such as death or hospitalization (2). Identification of predictors for the poor outcomes can be of benefit for risk stratification and clinical decision-making. In the HF process, the left atrium plays an integral part in the development of ventricular dysfunction and hemodynamic disorders through the reservoir, conduit, and booster pump phases (3). It has been proven that left atrial (LA) dysfunction has a predictive value in the long-term survival of patients with HF (4), which highlights the significance of evaluating LA mechanical function in clinical applications. LA strain derived from speckle tracking echocardiography (STE) is a relatively accurate indicator to reflect LA intrinsic function. Recently, the peak atrial longitudinal strain (PALS) of the left atrium has been applied for the identification of early LA impairment and the prediction of adverse events in patients with HF (5). In 2011, Helle-Valle et al. first demonstrated that LA strain could be a new non-invasive predictor of cardiovascular mortality or heart transplantation in patients with HF (6). Subsequently, a growing body of studies investigated the association between PALS and adverse clinical outcomes in both HF with preserved ejection fractions (HFpEF) and HF with reduced EF (HFrEF) (7–10). However, the results were inconsistent and most of the studies are single-center reports with relatively limited sample sizes. There is currently a lack of meta-analysis about the pooled effect of PALS on predicting the prognosis of patients with HF. Herein, we aimed to summarize the current pieces of evidence to determine whether PALS as a continuous variable might predict adverse events in patients with HF and if so, to estimate its effect size by conducting this systematic review and meta-analysis.

## Methods

### Search Strategy

This study was reported in adherence to Preferred Reporting Items for Systematic Reviews and Meta-analysis (PRISMA) statements. The PubMed, Embase, and Scopus databases were systematically searched from inception to 30 April 2022. The following keywords were used as search terms: “HF,” “atrial strain,” “atrial deformation,” and “atrial longitudinal strain.” References of included articles were manually searched to identify additional eligible studies. No language restrictions were applied. Specific search strategies for each database are detailed in Supplementary Table 1. Our original study design was registered prospectively in the PROSPERO database (registration number CRD42020185034).

### Eligibility Criteria

All studies were screened by two independent authors according to the following inclusion criteria: (1) studies that included adult patients with HF, (2) LA strain was measured on STE, (3) prospective or retrospective studies in which death and/or cardiac hospitalization were defined as endpoints, and (4) LA strain was used as a continuous variable to predict adverse outcomes during follow-up. Studies in which LA strain was evaluated using cardiac magnetic resonance imaging (MRI) were excluded because of technical differences. Case reports, reviews, letters, and editorials were also excluded. When multiple reports were derived from the same data set, only the most recent or complete study was enrolled in this review.

### EndPoint

The primary endpoint was defined as a composite outcome of all-cause death and cardiac hospitalization that varied from worsening HF, stroke, myocardial infarction, atrial fibrillation, and heart transplantation.

### Definitions

For the HF phenotype, we used the definitions from the 2016 European Society of Cardiology Guidelines (11). The diagnosis criterion for HFpEF was left ventricular ejection fraction (LVEF) ≥50%, HF with mid-range EF (HFmrEF) was LVEF 40–49%, and HFrEF was LVEF < 40%.

The peak positive longitudinal strain during the LA reservoir phase was defined as PALS, which was calculated by averaging the peak values of all LA segments from two- and four-chamber views or only four-chamber view as the european association of cardiovascular imaging (EACVI)/american society of echocardiography (ASE)/Industry Task Force recommended (12).

### Data Extraction and Quality Assessment

Two authors independently extracted data and summarized them in a data extraction file. Any disagreement was resolved by consensus or by consulting a third author. The missing data of eligible studies were attempted to obtain from the original authors by e-mail. The studies selected in our meta-analysis were evaluated for methodological quality using the Newcastle-Ottawa Scale (0–9 points) based on selection, comparability, and outcomes.

### Statistical Analysis

Data for continuous variables were pooled to calculate a weighted mean difference (WMD) and 95% confidence interval (CI). The WMD of PALS between patients with event and without event was separately computed and compared. The pooled hazard ratio (HR) and 95% CI of PALS per unit increase were calculated to evaluate their prognostic value for the primary endpoints. Forest plots were constructed to display overall effects using a random-effects model. The *I*^2^ measure was used to assess statistical heterogeneity among studies. *I*^2^ values of 25, 50, and 75% were represented as low, moderate, and high heterogeneity, respectively. We conducted a subgroup analysis to determine the impact of PALS on prognosis in patients with HFpEF, HFmrEF, and HFrEF. Sensitivity analysis was performed to exclude the effect of conference abstracts without complete published text on the overall pooled estimates. To assess publication bias, funnel plot and Egger’s test were chosen to examine the study distribution. The trim and fill analysis was further used to evaluate theoretical missing research studies because of publication bias. A bubble plot performed in R 4.0.2 displays the optimal cutoff value and area under the curve (AUC) from receiver operating characteristic (ROC) analysis in each study. RevMan 5.3 (Cochrane Collaboration, Oxford, United Kingdom) and Stata 14.0 (StataCorp, TX, United States) were used to perform statistical analysis with two-tailed *p*-values. A *p*-value of < 0.05 was considered statistically significant.

## Results

### Study Selection and Quality Assessment

**FIGURE 1:**
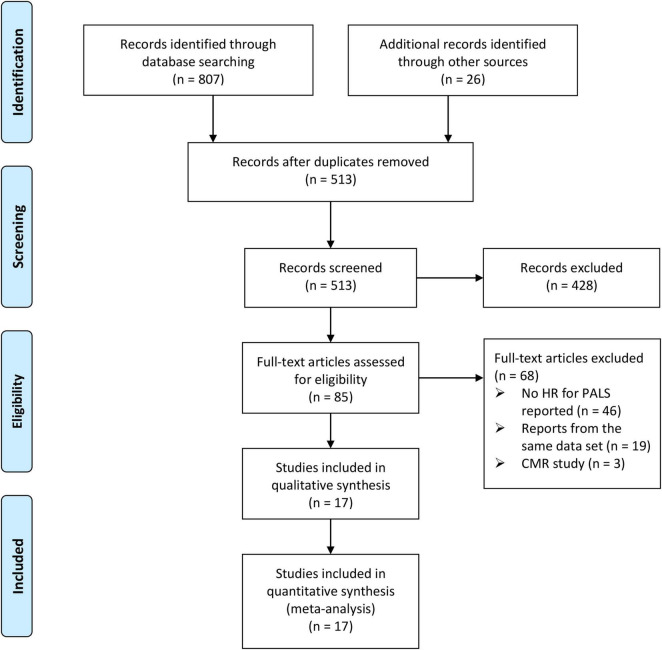
The Preferred Reporting Items for Systematic Reviews and Meta-analysis (PRISMA) flowchart of the study selection process.

From the initial 833 papers screened according to the search strategy and retrieved from reference lists, 513 papers remained following the elimination of duplicates. In total, 85 studies were identified as potentially eligible after screening the titles and abstracts. Following the full-text review, 68 studies were excluded due to not meeting the inclusion criteria. Consequently, a total of 17 studies that included 7,787 patients with HF were analyzed in the final analysis (4, 6–10, 13–23). The screening process and results are shown in Figure 1.

Methodological quality assessment using the Newcastle-Ottawa Scale showed high scores (7 points or more) in the 17 studies enrolled in our meta-analysis (Supplementary Table 2). The study object, patient population, and outcome were well defined in all studies. Five studies reported reproducibility for echocardiographic measurements, with the interobserver and intraobserver correlation coefficients of PALS varying from 0.85 to 0.98 and from 0.91 to 0.96, respectively (Supplementary Table 3).

### Patient Characteristics

Table 1 summarizes the characteristics of the 17 studies included in the systemic review. Among them, the majority (*n* = 13) were prospective cohort studies from single-center or multi-center. For the patient population, 4 studies reported only HFpEF, 1 reported only HFmrEF, 6 reported only HFrEF, and others reported mixed HF. The mean age ranged from 58 to 76 years, and 57.8% were men. Among the 11 studies that reported comorbidities, the most common diseases were hypertension (54.8%) and ischemic heart disease (39.0%). The mean baseline PALS among reported studies varied from 8.8 to 36.2%. During a follow-up time ranging from 3.3 to 60 months, 3,029 patients (38.9%) experienced the composite endpoint of all-cause death and cardiac hospitalization.

**TABLE 1:** Summary of study design and patient characteristics.

Publication	Design	HF (n)	Population	Inclusion criteria	Exclusion criteria	Etiology	Age (years)	Male (%)	HTN (%)	DM (%)	IHD (%)	LVEF (%)
Mandoli et al. (22)	Prosp	84	HFrEF	HF based on the ESC guidelines, LVEF <40%	Primary lung diseases, PH, CAD involving right heart, assist device implantation, heart transplantation, more than mild valvular stenosis	NR	60 ± 12	82	44	16	41	28 ± 5
Bouwmeester et al. (23)	Prosp	174	HFrEF, HFmrEF, HFpEF	Age ≥18 years, HF based on the ESC guidelines	Cardiothoracic surgery, pregnancy, severe renal failure, AF	NR	68 [59−75]	69	51	15	39	44 [34−49]
Rossi et al. (20)	Prosp	626	HFrEF	LVEF <40%	AF, heart surgery or transplantation, severe valvular disease, MI, malignancies	Ischemic, other causes	65 ± 11	78	39	49	55	30 ± 7
Bekki et al. (21)	NR	121	HFpEF	HFpEF	AF	NR	76 ± 14	60	NR	NR	NR	63 ± 8
Sciaccaluga et al. (13)	Prosp	118	HFrEF, HFmrEF, HFpEF	Age >18 years, *de novo* acute HF	Active cancer, poor echocardiographic window	Ischemic (62%), dilated (20%), heart valve disease (7%) and other causes (11%)	69 ± 12	75	NR	NR	62	33 ± 12
Park et al. (14)	prosp	3818	HFrEF, HFmrEF, HFpEF	All hospitalized patients with symptoms or signs HF	Patients with severe primary valvular heart disease or with ACS	NR	71 ± 14	53	58	34	33	40 ± 16
Deferm et al. (4)	Prosp	31	HFrEF	Age ≥18 years, presented with symptomatic decompensated HFrEF	PAWP <15 mmHg, cardiac index ≥2.6 l/min/m^2^, ventricular assist devices, mitral valve intervention	Ischemic (50%), non-ischemic (50%)	64 ± 15	77	48	29	48	20 ± 12
Vrettos et al. (15)	Prosp	134	HFmrEF, HFpEF	LVEF ≥50%, or LVEF of 40–49%	NR	NR	NR	NR	NR	NR	NR	NR
Malagoli et al. (7)	Prosp	286	HFrEF	18–85 years, sinus rhythm, pharmacologic therapy for ≥30 days	Primary valve disease, mechanical valve prosthesis, significant comorbid illness	Ischemic, other causes	67 ± 11	81	69	25	64	32 ± 6
Bolog et al. (16)	Prosp	182	HFpEF	Non-acute HFpEF	ACS, severe valvular disease, arrhythmia, cardiomyopathies, class IV NYHA	NR	65 ± 11	48	NR	NR	NR	NR
Stone et al. (17)	Retro	944	HFmrEF	LVEF 40–50%	AF, severe arrhythmia, moderate or severe valve disease, prosthetic valves, greater than mild pericardial effusion, cancer on chemotherapy	NR	NR	NR	NR	NR	NR	NR
Saha et al. (18)	NR	49	HFrEF	HFrEF in sinus rhythm	NR	NR	72 ± 13	58	12	8	68	31 ± 8
Carluccio et al. (8)	Prosp	405	HFrEF	HFrEF in sinus rhythm	HF because of a reversible cause, hospital readmission for worsening HF in the last month, HCM, untreated thyroid disease, pericardial disease, amyloidosis, prosthetic valve, recent MI (≤6 months), <1-year life expectancy	hypertensive (19%), ischemic (38%), idiopathic (33%), other causes (10%)	65 ± 12	76	19	26	38	30 [25–35]
Lofrano-Alves et al. (19)	NR	51	HF	New or worsening HF symptoms and need of intravenous therapy	NR	NR	58 ± 12	59	NR	NR	NR	31 ± 10
Santos et al. (9)	Prosp	357	HFpEF	Symptomatic HF, LVEF ≥45%, controlled systolic blood pressure, serum potassium level <5 mmol/L	Insufficient imaging quality	NR	69 ± 10	43	93	42	30	60 ± 8
Freed et al. (10)	Prosp	308	HFpEF	Age ≥21 years, LVEF ≥50%, presence of HF as defined by Framingham criteria.	Severe valvular disease, cardiac transplantation, LVEDV >97 mL/m^2^, constrictive pericarditis	NR	65 ± 13	36	75	30	50	61 ± 6
Helle-Valle et al. (6)	Prosp	99	HF	Ischemic or dilated cardiomyopathy (NYHA II-IV)	NR	ischemic or dilated cardiomyopathy	NR	NR	NR	NR	NR	NR

**Publication**	**PALS%**	**ECG-gating**	**Chamber view**	**Software**	**Definition of endpoints**	**Follow-up, month**	**Events,%**

Mandoli et al. (22)	14 ± 4.6	R-R	Two and four	Echopac	Cardiac death and HF hospitalization	42 ± 3.6	57
Bouwmeester et al. (23)	27 [20–35]	R-R	Two and four	QLAB 13	All-cause death and HF hospitalization	12	13
Rossi et al. (20)	16 ± 8	R-R	Two and four	EchoPAC or QLAB	All-cause death and HF hospitalization	NR	42
Bekki et al. (21)	17.8 ± 9.9	NR	NR	TOMTEC	HF hospitalization	10.6 ± 9.0	27
Sciaccaluga et al. (13)	18.1 ± 13.6	R-R	Two and four	Echopac	All-cause death	8.1	23.7
Park et al. (14)	14.7 ± 10.1	R-R	Two and four	TomTec-Arena v4.6	All-cause death and HF hospitalization	30.6 [11.6–54.4]	52.8
Deferm et al. (4)	8.80 ± 3.0	R-R	Four	Image arena v4.6	All-cause death and HF hospitalization	21.8 ± 9.6	61.3
Vrettos et al. (15)	NR	NR	NR	NR	HF hospitalization	57 (range 11.1)	8
Malagoli et al. (7)	19.4 ± 9.4	R-R	Two and four	EchoPAC v112	MACE (HF hospitalization, MI, stroke, and cardiac death)	48 ± 11	34
Bolog et al. (16)	NR	NR	NR	NR	Cardiac death, ACS, worsening HF, AF, stroke	20 [18–26]	26.9
Stone et al. (17)	NR	NR	NR	QLAB	All-cause death	60	2.2
Saha et al. (18)	11 ± 6	R-R	Four	Echopac v13	All-cause death and HF hospitalization	32 ± 9	48.0
Carluccio et al. (8)	15.5 [11.2–20.6]	R-R	Two and four	Echopac v113	All-cause death and HF hospitalization	29.6 [13.1–51.3]	34
Lofrano-Alves et al. (19)	9.7 ± 5.5	NR	NR	NR	Cardiac death, heart transplantation, circulatory assist device use or readmission	3.3 ± 2.1	54
Santos et al. (9)	25.9 ± 7.7	R-R	Two and four	TomTec Imaging Systems	Cardiac death, HF hospitalization, aborted sudden death	31 [18–43]	25.5
Freed et al. (10)	36.2 ± 14.9	R-R	Two and four	TomTec v4.5	Cardiac hospitalization or death	13.8 [4.5–23.9]	37
Helle-Valle et al. (6)	NR	NR	NR	NR	All-cause death or heart transplantation	36	28

*ACS, acute coronary syndrome; AF, atrial fibrillation; CAD, coronary artery disease; DM, diabetes mellitus; HCM, hypertrophic cardiomyopathy; HF, heart failure; HFmrEF, heart failure with mid-range ejection fraction; HFpEF, heart failure with preserved ejection fraction; HFrEF, heart failure with reduced ejection fraction; HTN, hypertension; IHD, ischemic heart disease; LVEF, left ventricular ejection fraction; MACE, major adverse cardiac event; MI, myocardial infarction; NR, not reported; NYHA, New York Heart Association; PALS, peak atrial longitudinal strain; PAWP, pulmonary artery wedge pressure; PH, pulmonary hypertension; Prosp, prospective; Retro, retrospective.*

### Relationship Between Peak Atrial Longitudinal Strain and Events

**FIGURE 2:**
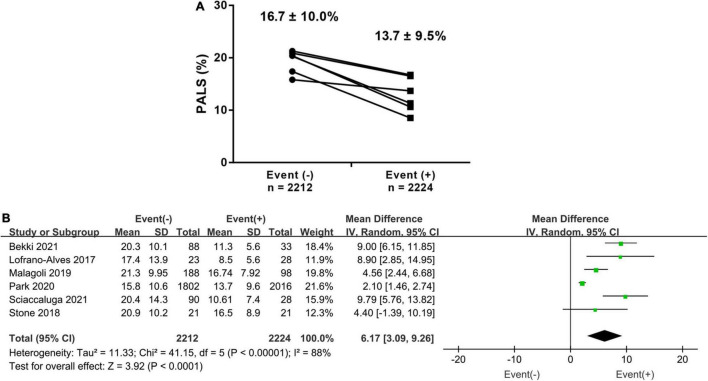
**(A)** Baseline difference (mean ± SD) of PALS in heart failure (HF) patients with endpoints and those without endpoints. **(B)** The forest plot shows a weighted mean difference and 95% CI of baseline peak atrial longitudinal strain (PALS) between the above two groups.

In total, 6 studies investigated the difference in PALS in HF patients with and without events. The PALS value for patients experiencing events was 13.7 ± 9.5%, while for those without events, it was 16.7 ± 10.9%. The pooled results showed that there was a 6.17% (95% CI 3.09–9.26, *p* < 0.001, *I*^2^ = 88%) difference in PALS between the two groups, as shown in Figures 2A,B. Subgroup analysis based on LVEF phenotype also showed similar results to the overall analysis. The patients with events had markedly lower PALS than those without events in the HFpEF, HFmrEF, and HFrEF subgroups (Supplementary Figure 1).

### Prognostic Value of Peak Atrial Longitudinal Strain for EndPoint

**FIGURE 3:**
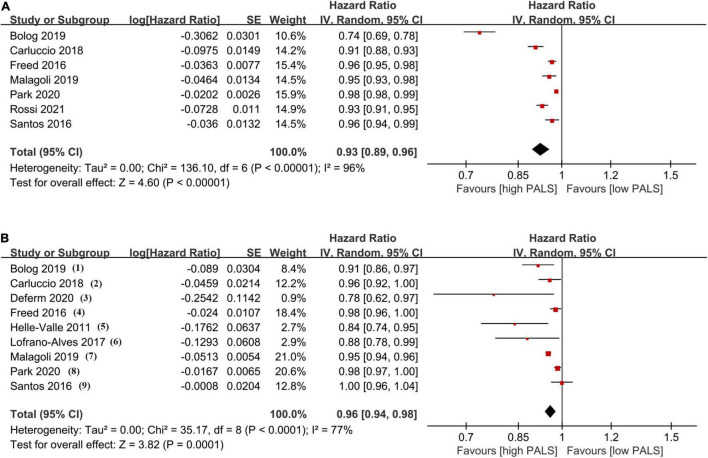
The pooled hazard ratio (HR) of peak atrial longitudinal strain (PALS; per 1-unit increase) in univariate analysis **(A)** and multivariate analysis **(B)** for predicting endpoint. Covariates in multivariate analysis: (1) age, hypertension, diabetes, left atrial volume index (LAVI), left ventricular global longitudinal strain (LVGLS); (2) eplerenone in mild patients hospitalization and survival study in heart failure (EMPHASIS-HF) risk score, New York Heart Association (NYHA) class, log(BNP), implanted cardioverter defibrillator (ICD) at baseline, cardiac resynchronization therapy (CRT) implant during follow-up, LAVI, end-diastolic volume index (EDVI), left ventricular ejection fraction (LVEF), E/e’, mitral regurgitation severity, LVGLS; (3) LAVI, left ventricular end-diastolic volume (LVEDV), and LVGLS; (4) sex, atrial fibrillation (AF), meta-analysis global group in chronic heart failure (MAGGIC) risk score, LV mass, LAV, E/e’, LVGLS, RV free wall strain; (5) age, N-terminal pro-brain natriuretic peptide (NTproBNP), LA area, LVEF, E/e’; (6) LVEF, LVGLS, right ventricular global longitudinal strain (RVGLS); (7) age, NYHA class, glomerular filtration rate (GFR), brain natriuretic peptide (BNP), left ventricular end-systolic volume index (LVESVi), LVEF, LAVI, E/A, and E/e’; (8) age, sex, BMI, NYHA IV, diastolic blood pressure (DBP), heart rate, hypertension, diabetes, AF, ischemic heart disease (IHD), hemoglobin, creatinine, total cholesterol, LVEF, log(NTproBNP), and LAVI; (9) age, sex, race, randomization strata, enrollment region, randomized treatment assignment, AF, heart rate, NYHA, stroke, creatinine, hematocrit, LVEF, LAVI, and LVGLS.

The association between PALS and the incidence of endpoints in univariate and multivariate Cox models is provided in Figure 3. In univariate analysis, the results from 7 studies indicated a significantly decreased HR (0.93, 95% CI 0.89–0.96, *p* < 0.001, *I*^2^ = 96%) for PALS (per 1-unit increase). Importantly, combining all multivariate HRs after adjustment in 9 studies, each unit increase of PALS contributed to a 4% (HR 0.96, 95% CI 0.94–0.98, *p* < 0.001, *I*^2^ = 77%) risk reduction for the primary endpoint. In subgroup analysis based on LVEF level, pooled HR in the univariate Cox model was consistent with those found in the overall analysis (Supplementary Figure 2).

**FIGURE 4:**
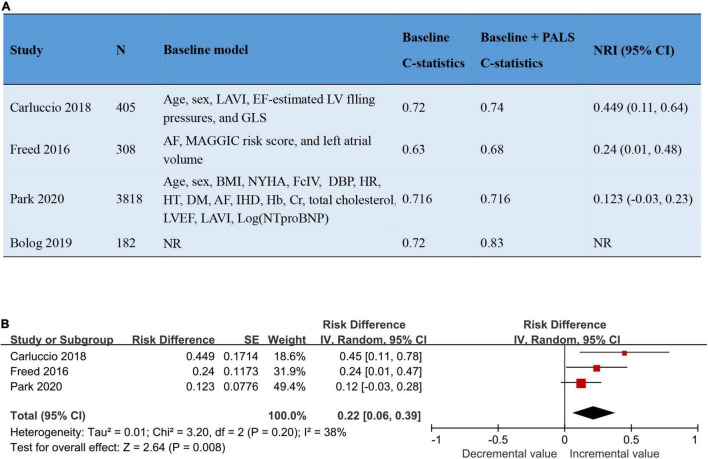
The addition of PALS to baseline risk models led to a significant improvement in the predictive power of models. **(A)** Four studies reported the addition of PALS to baseline risk models. **(B)** The forest plot shows the pooled NRI of PALS for predicting adverse outcomes. AF, atrial fibrillation; BMI, body mass index; Cr, creatinine; DBP, diastolic blood pressure; DM, diabetes mellitus; Hb, hemoglobin level; GLS, global longitudinal strain; HR, heart rate; HT, hypertension; IHD, ischemic heart disease; LAVI, left atrial volume index; LVEF, left ventricular ejection fraction; NRI, net reclassification index; NR, not reported; NYHA, New York Heart Association.

**FIGURE 5:**
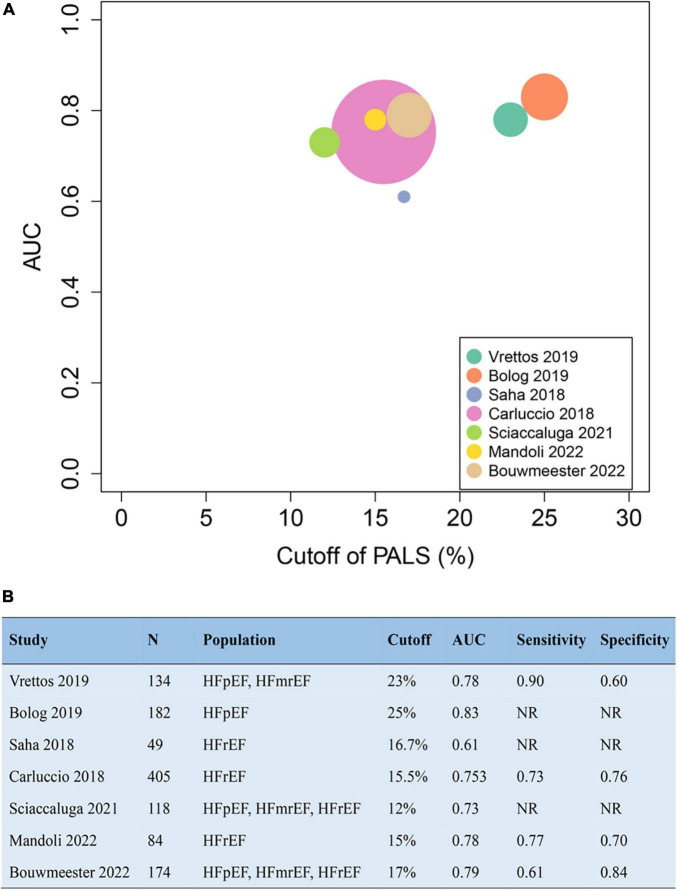
The cutoff value and AUC of peak atrial longitudinal strain (PALS) in receiver operating characteristics curve to identify risk stratification for endpoint **(A)** according to the seven studies **(B)**. AUC, area under the curve.

The addition of PALS to baseline risk models contributed to the increase of both the C-statistic and the net reclassification index (NRI), varying from 0 to 0.11 and from 0.12 to 0.45, respectively. The pooled NRI of PALS for predicting adverse outcomes based on 3 studies was 0.22 (95% CI 0.06–0.39, *p* = 0.008, *I*^2^ = 38%). Details about the predictive increment of PALS in the Cox hazard models are summarized in Figure 4. On the basis of the ROC curve analysis available in 7 studies, the ability of PALS to identify risk stratification is graphically presented in Figure 5. The cutoff value and AUC ranged from 12 to 25% and from 0.61 to 0.83, respectively. Furthermore, we compared PALS prognostic cutoff values between patients with HFpEF and HFrEF. As a result, patients with HFpEF or HFmrEF had a higher PALS cutoff value (ranging from 2 to 25%) according to studies by Vrettos and Blog et al., while patients with HFrEF presented a lower PALS cutoff value (ranging from 15 to 16.7%) based on the studies of Saha et al.

### Sensitivity Analysis

After excluding conference abstracts without complete published texts, the sensitivity analysis did not reveal a significant change in the results of the overall analysis (Supplementary Figure 3). In addition, sensitivity analyses were carried out for prospective studies and studies reporting longer than the 1-year follow-up, respectively, which showed consistent results to the overall analysis (Supplementary Figures 4, 5). These suggested that the overall results were stable and reliable.

### Publication Bias

Although visual inspection of the funnel plot suggested some asymmetry at the bottom, Egger’s test (*p* = 0.218) revealed no evidence of publication bias in the 9 studies reporting HR in the multivariable Cox model (Supplementary Figure 6). Additionally, trim and fill analysis indicated no effect of publication bias on the overall estimate (no trimming performed and data unchanged), as shown in Supplementary Figure 7.

## Discussion

To our knowledge, this meta-analysis is the first systematic collection and quantitative synthesis of reported evidence on the prognostic value of PALS for patients with HF. The key finding was that PALS was an independent predictor for all-cause mortality and cardiac hospitalization. Moreover, this finding was generally consistent regardless of HF phenotypes based on LVEF. The addition of PALS could significantly improve the predictive power of conventional risk models. Collectively, our findings emphasize the clinical importance of evaluating LA deformation in the prognosis of HF.

In the disease progression of HF, the continuous dynamic interplay between atrial and ventricular mechanics plays an important role in cardiac performance. LA anatomical and functional alterations are the results of volume and pressure overload caused by left ventricular changes (5). LA decompensation and remodeling can further aggravate abnormalities in LV performance (24). In addition, intrinsic atrial myopathy, defined as decreased LA reservoir strain, may occur disproportionately to LV dysfunction and finally contribute to LA failure (25). It has been proven that LA reservoir function is associated with the extent of atrial fibrosis and chamber stiffness prior to LA geometrical remodeling (26). PALS, as a simple, feasible, and non-invasive parameter to represent LA reservoir function, was also correlated well with invasive LV filling pressure (27). The strong internal relationship between reduced PALS alongside impaired cardiac performance might explain why PALS is a strong and independent predictor of adverse clinical outcomes in HF. Indeed, PALS has been considered a sensitive prognostic marker in many clinical settings, such as chronic renal disease, hypertension, and cardiotoxicity (28).

This meta-analysis demonstrated that PALS showed meaningful prognostication in all clinical HF phenotypes (HFrEF, HFmrEF, and HFpEF) based on LVEF. In this regard, the results appeared to be widely representative and applicable to a broad spectrum of patients with HF. LVEF is essential to phenotype and guides the treatment of patients with HF, with an inverse correlation to mortality (29). However, no further trend was found to reflect prognosis when LVEF was above 40% (30). In addition, LVEF, dependent on afterload and preload, reflects the change in LV chamber size but not myocardial contractility (31). The inherent limitations of LVEF may restrict its ability to detect mild myocardial impairment and predict the gradation of risk in HFpEF. Recently, LA mechanics has attracted increasing attention for improving the diagnostic and prognostic evaluation of HF. PALS is a sensitive marker for diastolic assessment in patients at risk of HF and a good addition to conventional diastolic parameters (32). In the current grading of diastolic dysfunction, PALS could change progressively with the severity of diastolic dysfunction (33). The use of PALS in place of left atrial volume index (LAVI) may contribute to a more accurate categorization of patients with indeterminate diastolic function (32). Moreover, PALS could provide prognostic significance independent of clinical and echocardiographic assessment, even in patients with HFpEF or normal LA size (32, 34). Our meta-analysis further underscored the additional prognostic implication of evaluating LA mechanics in clinical management, overcoming the limitations of LVEF.

Previously, several multivariable risk models based on demographic, biomarker, and imaging indicators have been developed to understand HF prognosis (30, 35, 36). A few independent and routine risk makers have emerged for the prediction of mortality, such as age, renal function, blood pressure, LVEF, brain natriuretic level, and New York Heart Association (NYHA) class. However, these current models only had moderate clinical applicability for the prediction of death, and their discriminative ability to predict hospitalization or the composite outcome of death and hospitalization appeared to be even poorer (35, 36). Thus, risk prediction in HF remains difficult, as important prognosticators for hospitalization are lacking. Recently, Molnár et al. proposed that PALS assessment may help not only to identify subtle cardiac impairment but also to update the current disease grading and risk scores (28). Indeed, our meta-analysis found that the addition of PALS to current models contributed to significant improvement in the risk prediction of all-cause death and hospitalization, indicating that PALS could be considered a routine measurement for evaluating clinical prognosis in patients with HF. The establishment of a more effective prognosis model will be useful in identifying HF patients with adverse events on currently recommended treatment and further developing more targeted therapy strategies to improve outcomes for high-risk patients.

Our meta-analysis revealed that patients with HF experiencing events presented worse PALS than those without events. The cutoff value of PALS for identifying high-risk patients has attracted much attention and has not yet been determined. We provided a reference range of PALS cutoff value, from 12 to 25%, based on the available pieces of literature. Notably, patients with HFpEF or HFmrEF seemed to have higher PALS cutoff values when compared with those with HFrEF. To our knowledge, such a comparison has not yet been reported. According to Park et al., an ordinal decrease of PALS at baseline was detected in patients with HFpEF, HFmrEF, and HFrEF (14). Whether PALS thresholds for prognosis differ across HF subgroups is an area for further investigation. Our result provides new ideas for identifying ideal PALS thresholds in HF for follow-up studies.

Our study has some limitations. First, this is a meta-analysis of observational studies. Inevitably, multiple variations in the clinical settings, population characteristics, follow-up time, and endpoint definition were all possible sources of heterogeneity across different studies. We used a random-effects model to correct for these variations. Subgroup analyses and sensitivity analyses were performed to analyze and eliminate the high heterogeneity in our study. Although heterogeneity still existed, our findings remained significant, implicating that the prognostic value of PALS appears to be suitable for a broad range of patients with HF. Second, the funnel plot revealed that some asymmetry due to a small number of enrolled studies reduced the power of this test. Third, while LV global longitudinal strain (GLS) was associated with adverse outcomes in patients with HF based on recent reports (31), the small number of studies reporting GLS in prognosis did not allow for a comparison of predictive ability between PALS and GLS in our meta-analysis. Fourth, the absence of standardization among multiple software programs makes it difficult to unify the quality of the imaging. However, inter- and intraobserver correlation according to reported studies suggested high reproducibility of PALS measurements. Last, because individual patient data from original studies were not available, we cannot define the cutoff value of PALS for identifying high-risk patients and its diagnostic accuracy in ROC curves. Therefore, high-quality evidence in large prospective cohorts is warranted to determine the best diagnostic threshold for prognosis.

## Conclusion

In this meta-analysis, PALS was found to be an independent predictor for all-cause mortality and cardiac hospitalization in patients with HF. The addition of PALS to conventional risk models could improve the predictive power for clinical endpoints. Overall, these findings indeed emphasize the clinical application of LA mechanics by STE to detect high-risk patients and predict prognosis.

## Data Availability Statement

The original contributions presented in this study are included in the article/Supplementary Material, further inquiries can be directed to the corresponding author.

## Author Contributions

FJ and WC: conceptualization. FJ, AC, and LF: study selection. FJ, AC, WC, and DZ: formal analysis. FJ and DZ: statistical analysis. FJ, AC, DZ, and LF: drafting of the manuscript. WC, DZ, and LF: critical revision of the manuscript. WC: funding acquisition. All authors contributed to the article and approved the submitted version.

## Conflict of Interest

The authors declare that the research was conducted in the absence of any commercial or financial relationships that could be construed as a potential conflict of interest.

## Publisher’s Note

All claims expressed in this article are solely those of the authors and do not necessarily represent those of their affiliated organizations, or those of the publisher, the editors and the reviewers. Any product that may be evaluated in this article, or claim that may be made by its manufacturer, is not guaranteed or endorsed by the publisher.
