# Supplementary material for: Prognostic Value of Left Atrial Strain in Heart Failure: A Systematic Review and Meta-Analysis
Source: Front Cardiovasc Med. 2022 Jul 1;9:935103. doi: 10.3389/fcvm.2022.935103 (PMC9283726; doi:10.3389/fcvm.2022.935103)
Supplement: Supplementary file 1 [file Data_Sheet_1.docx]

Supplementary Material

# Supplementary Tables

## Table S1. Complete search strategy for each database

| Database | Search strategy | Articles searched |
| --- | --- | --- |
| PubMed | ((heart failure[Title/Abstract]) AND (((atrial strain[Title/Abstract]) OR atrial deformation[Title/Abstract]) OR atrial longitudinal strain[Title/Abstract])) | 176 |
| Embase | 1 'atrial strain':ab,ti OR 'atrial deformation':ab,ti OR 'atrial longitudinal strain':ab,ti  2 'heart failure':ab,ti  1 and 2 | 414 |
| Scopus | (TITLE-ABS-KEY ("atrial strain" OR "atrial deformation" OR "atrial longitudinal strain") AND TITLE-ABS-KEY ("heart failure")) | 217 |

## Table S2. Methodological quality assessment using the Newcastle-Ottawa Scale for included studies

| **Publication** | **Selection** | | | | **Comparability** | **Outcome** | | | **Total score** |
| --- | --- | --- | --- | --- | --- | --- | --- | --- | --- |
|  | **S1** | **S2** | **S3** | **S4** | **C** | **O1** | **O2** | **O3** |  |
| Mandoli et al | **🟑** | **🟑** | **🟑** | **🟑** | **🟑** | **🟑** | **🟑** | **🟑** | 8 |
| Bouwmeester et al | **🟑** | **🟑** | **🟑** | **🟑** | **🟑** | **🟑** | **🟑** | **🟑** | 8 |
| Rossi et al | **🟑** | **🟑** | **🟑** | **🟑** | **🟑** | **🟑** | **🟑** |  | 7 |
| Bekki et al | **🟑** | **🟑** | **🟑** | **🟑** | **🟑** | **🟑** |  | **🟑** | 7 |
| Sciaccaluga et al | **🟑** | **🟑** | **🟑** | **🟑** | **🟑** | **🟑** | **🟑** | **🟑** | 8 |
| Park et al | **🟑** | **🟑** | **🟑** | **🟑** | **🟑** | **🟑** | **🟑** | **🟑** | 8 |
| Deferm et al | **🟑** | **🟑** | **🟑** | **🟑** | **🟑** | **🟑** | **🟑** | **🟑** | 8 |
| Vrettos et al | **🟑** | **🟑** | **🟑** | **🟑** | **🟑** | **🟑** | **🟑** | **🟑** | 8 |
| Malagoli et al | **🟑** | **🟑** | **🟑** | **🟑** | **🟑** | **🟑** | **🟑** | **🟑** | 8 |
| Bolog et al | **🟑** | **🟑** | **🟑** | **🟑** | **🟑** | **🟑** | **🟑** | **🟑** | 8 |
| Stone et al | **🟑** | **🟑** | **🟑** | **🟑** | **🟑** | **🟑** | **🟑** |  | 7 |
| Saha et al | **🟑** | **🟑** | **🟑** | **🟑** | **🟑** | **🟑** | **🟑** | **🟑** | 8 |
| Carluccio et al | **🟑** | **🟑** | **🟑** | **🟑** | **🟑** | **🟑** | **🟑** | **🟑** | 8 |
| Lofrano-Alves et al | **🟑** | **🟑** | **🟑** | **🟑** | **🟑** | **🟑** |  | **🟑** | 7 |
| Santos et al | **🟑** | **🟑** | **🟑** | **🟑** | **🟑** | **🟑** | **🟑** | **🟑** | 8 |
| Freed et al | **🟑** | **🟑** | **🟑** | **🟑** | **🟑** | **🟑** | **🟑** | **🟑** | 8 |
| Helle-Valle et al | **🟑** | **🟑** | **🟑** | **🟑** | **🟑** | **🟑** | **🟑** | **🟑** | 8 |

## Table S3. Intraobserver and interobserver reproducibility for PALS

| Publication | Method | Intraobserver variability | Interobserver variability |
| --- | --- | --- | --- |
| Deferm et al | ICC | NR | 0.98 |
| Malagoli et al | Mean difference | 2.4 ± 1.1% | 3.5 ± 1.4% |
| Carluccio et al | ICC | 0.91 | 0.87 |
| Santos et al | ICC | 0.96 | NR |
| Freed et al | ICC | 0.94 | 0.85 |

ICC = intraclass correlation coefficient; NR = not reported.

# Supplementary Figures

## Figure S1


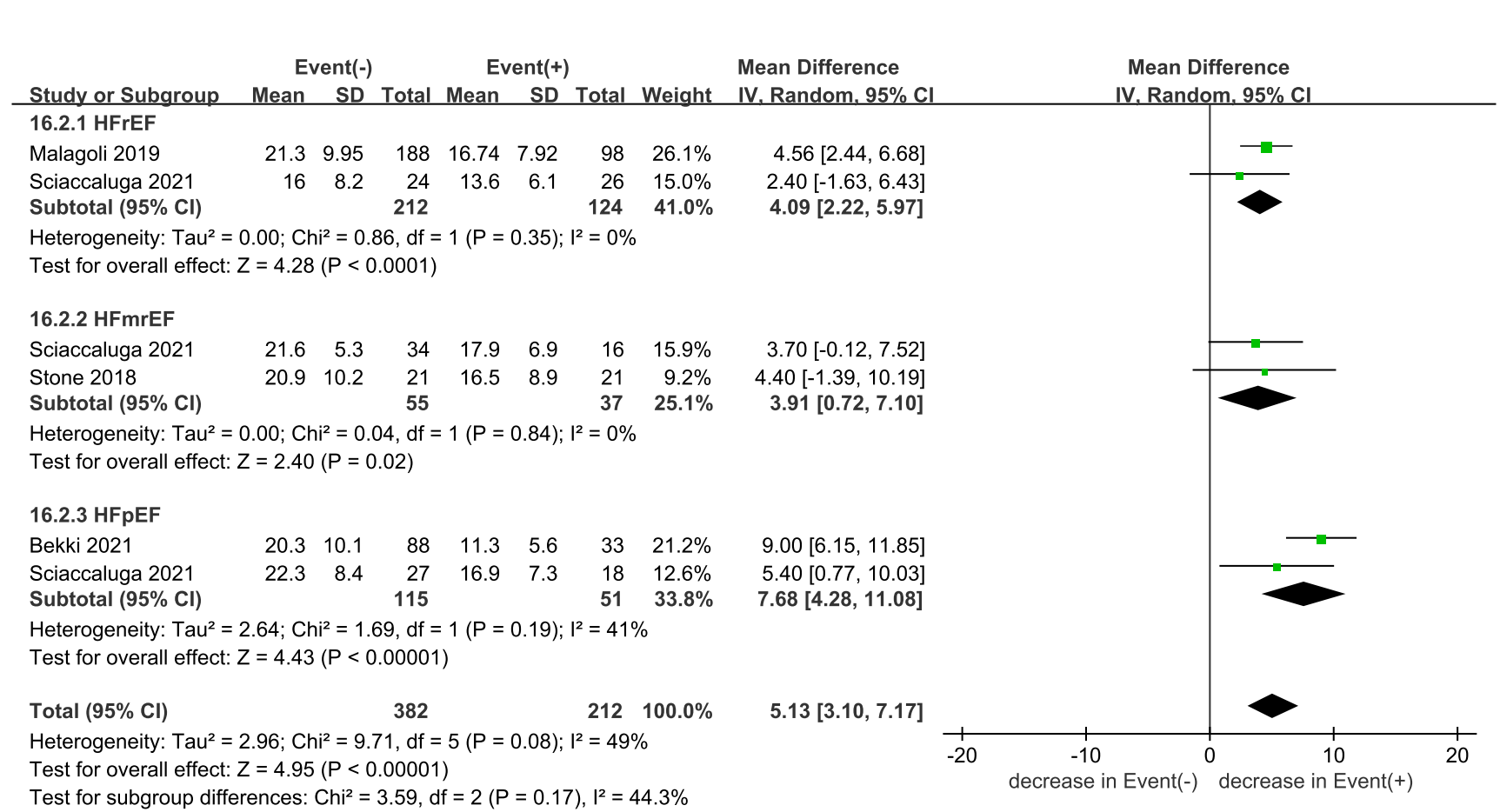


**Figure S1.** Subgroup analysis based on the LVEF phenotype revealed baseline differences in PALS in HF patients with and without endpoints.

## Figure S2


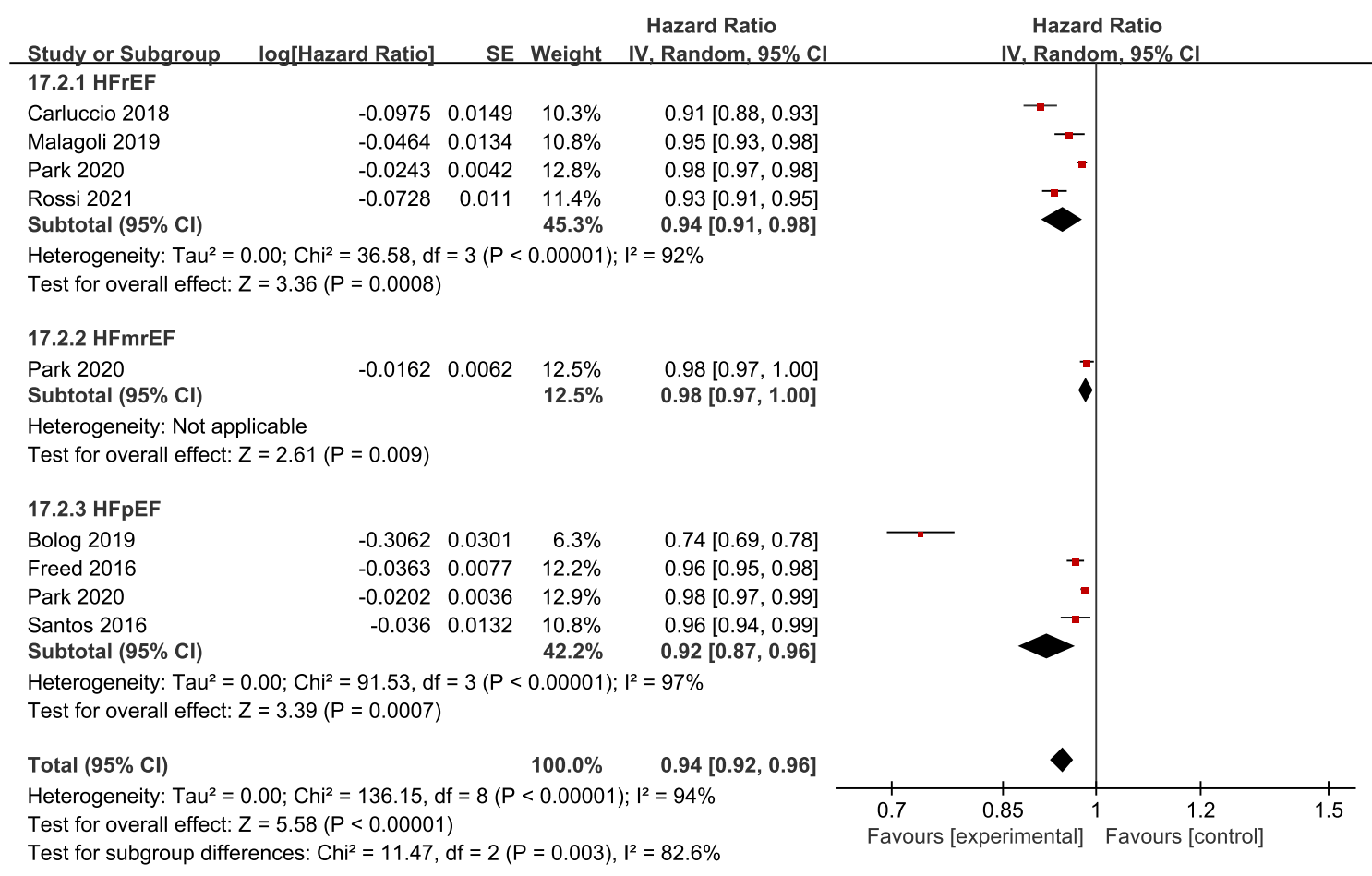


**Figure S2.** Subgroup analysis based on LVEF phenotype displayed summary HR of PALS (per 1-unit increase) in univariate analysis.

## Figure S3


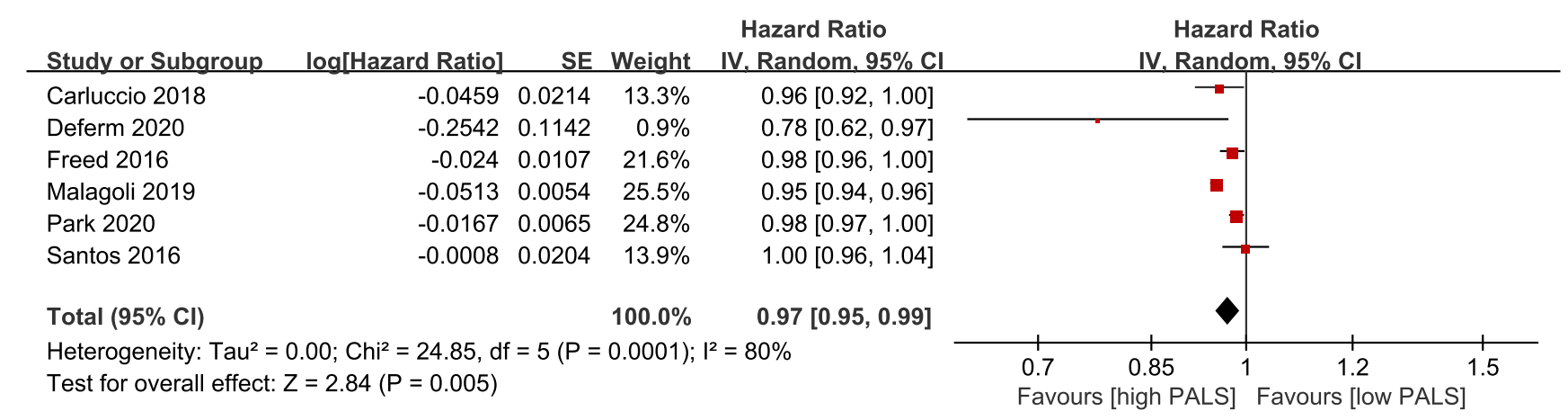


**Figure S3.** Sensitivity analysis of PALS in the multivariable model by excluding conference abstracts without complete published texts.

## Figure S4


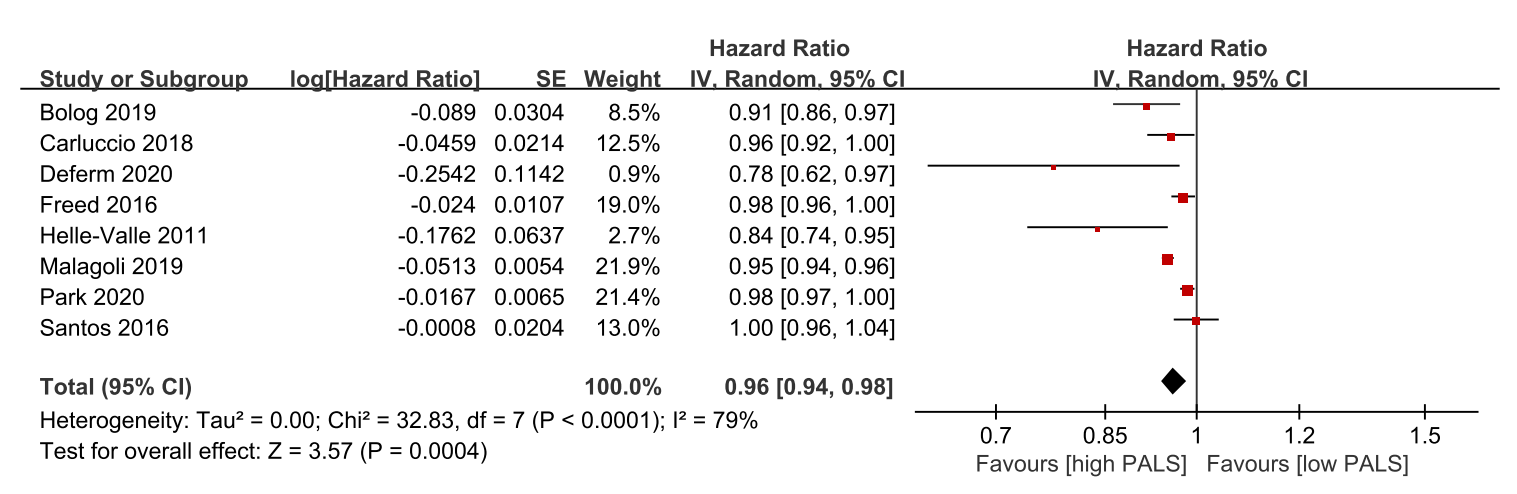


**Figure S4.** Sensitivity analysis of PALS in the multivariable model for prospective studies.

## Figure S5


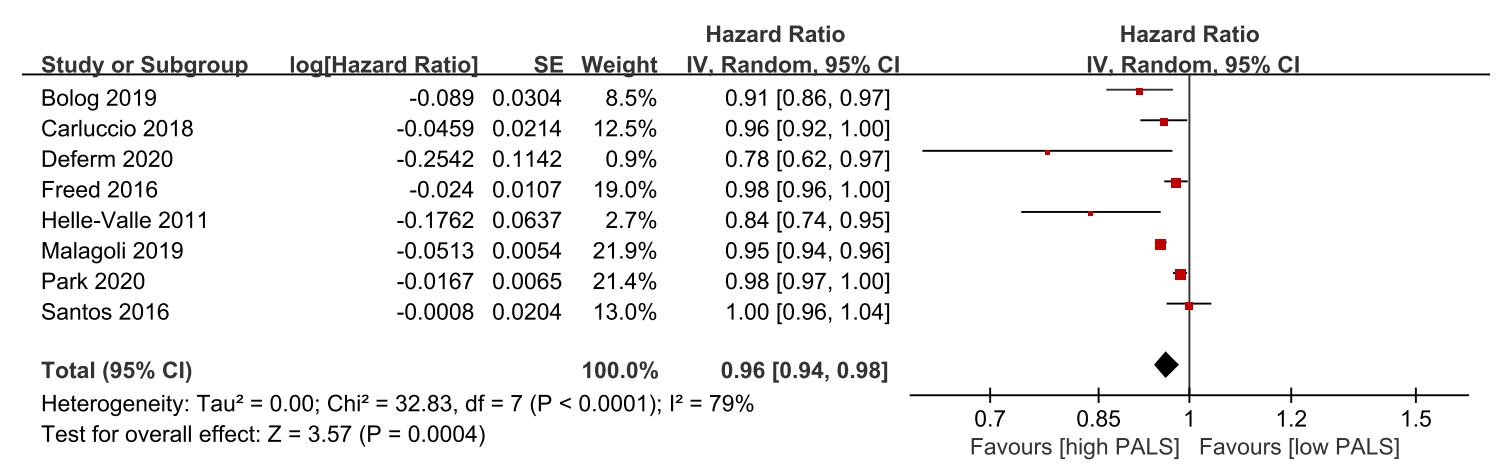


**Figure S5.** Sensitivity analysis of PALS in the multivariable model by excluding studies with less than 1-year follow up.

## Figure S6


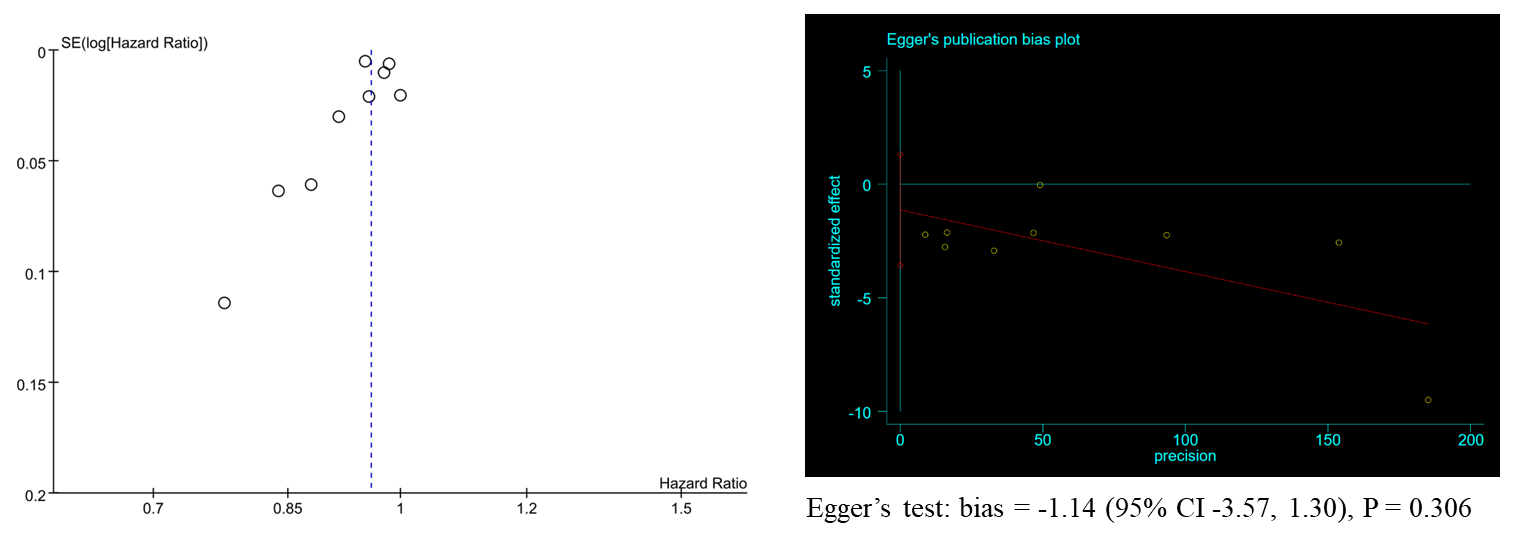


**Figure S6.** The funnel plot and Egger’s test were used to assess publication bias for PALS in the 10 studies that reported HRs in the multivariable model.

## Figure S7


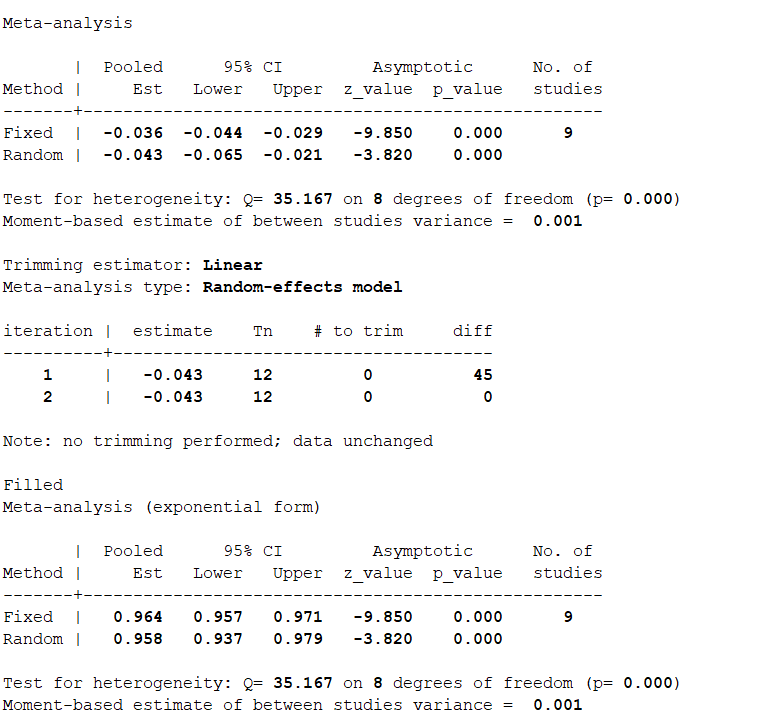


**Figure S7.** The trim and fill analysis indicated no theoretical missing studies.
